# From Physiology to Clinical Practice in Pancreatic Cancer-Related Thromboembolism—A Review

**DOI:** 10.3390/cancers18060890

**Published:** 2026-03-10

**Authors:** Monika Jarowicz, Michał Sekuła, Wiktor Kociemba, Katarzyna Polak, Joanna Taczała, Kamila Krupa, Hanna Miski, Marta Fudalej, Andrzej Deptała, Anna Badowska-Kozakiewicz

**Affiliations:** 1 Students’ Scientific Organization of Cancer Cell Biology, Department of Oncology Propaedeutics, Medical University of Warsaw, 01-445 Warsaw, Poland; monika.jarowicz@uckwum.pl (M.J.); s082692@student.wum.edu.pl (M.S.); s085113@student.wum.edu.pl (W.K.); s084636@student.wum.edu.pl (K.P.); s085346@student.wum.edu.pl (J.T.); kamila.krupa@student.wum.edu.pl (K.K.); s090695@student.wum.edu.pl (H.M.); 2 Department of Oncology Propaedeutics, Medical University of Warsaw, 01-445 Warsaw, Poland; marta.fudalej@wum.edu.pl (M.F.); andrzej.deptala@wum.edu.pl (A.D.); 3 Department of Oncology, National Medical Institute of the Ministry of the Interior and Administration, 02-507 Warsaw, Poland

**Keywords:** thromboembolism, cancer, pancreas, PDAC

## Abstract

Pancreatic cancer (PC) is a highly lethal malignancy linked to the highest rate of thromboembolic complications (TEC) among all solid tumors. TECs occur in approximately 5–40% of PC patients. The most common type of TEC in PC is venous thromboembolism (VTE). The mechanisms leading to frequent TEC in PC are complex and involve interactions between tumor-derived procoagulant factors and the prothrombotic tumor microenvironment (TME). Secretion of tissue factor and proinflammatory cytokines by tumor cells and the TME, overexpression of heparanase and podoplanin, impaired fibrynolysis and increased neutrophil extracellular trap formation lead to platelet hyperactivation resulting in hypercoagulability in PC. Understanding these mechanisms is crucial for identifying risk factors of TEC. Current thromboembolism risk models have limited predictive accuracy, which reduces their clinical usefulness. Identifying patients with thromboembolism is challenging because these events are often asymptomatic and their clinical presentation varies depending on the location of the thrombus. Treatment of VTE in PC depends on the phase of the VTE; in the acute phase, treatment primarily involves LMWH. For long-term management, LMWH may be replaced by direct oral anticoagulants such as apixaban, edoxaban, or rivaroxaban. In cases of VTE recurrence, increasing the LMWH dose, switching to an oral anticoagulant, or placing an inferior vena cava filter should be considered. LWMH and unfractionated heparin (UFH) are preferred options for VTE prophylaxis. Novel therapies, including factor XI inhibitors, show efficacy comparable to LMWH while offering a better safety profile.

## Simple Summary

Pancreatic cancer (PC) is a common and highly lethal solid tumor with rising incidence and mortality rates. The most common type of PC is pancreatic ductal adenocarcinoma, which has the highest incidence of thromboembolic complications of all malignant tumors. These events, particularly deep vein thrombosis and pulmonary embolism, are associated with increased mortality during the course of the disease. This review summarizes tumor- and microenvironment-driven mechanisms of thromboembolism in PC and highlights key risk factors related to the disease, patient characteristics, and treatment modalities. It also outlines warning signs that require further diagnostic evaluation and summarizes diagnostic methods, treatment options, and preventive measures. Finally, the review identifies deficiencies in the management of thromboembolic complications in PC patients and presents ongoing research aimed at improving patient care.

## 1. Introduction

Pancreatic cancer (PC) is one of the most prevalent and fatal malignancies in the world. In 2024, the estimated incidence of PC cases in the United States was 66,440, comprising 34,530 cases in men and 31,910 cases in women [1]. PC ranks as the 12 th most common cancer worldwide and, due to its poor prognosis, it is the sixth leading cause of cancer-related deaths in 2022 [2]. Trends indicate an increase in PC incidence and mortality, particularly among women, in the population aged 50 years and above, and in countries with a very high and high human development index, a summary indicator based on levels of education, national income, and life expectancy [3].

Pancreatic ductal adenocarcinoma (PDAC) is responsible for 90% of all cases of PC and exhibits the highest incidence of venous thromboembolism (VTE) complications in comparison to other types of solid tumor malignancies [4,5]. The incidence of thromboembolic events in PC can be up to four times higher than in other neoplasms, ranging from 5.4 to 41% [6,7,8,9]. The most prevalent cancer-related thromboembolic complications (TEC) are deep venous thrombosis (DVT) and pulmonary embolism (PE) [5]. Other complications include: thrombophlebitis migrans, also known as Trousseau syndrome, splenic vein thrombosis, spontaneous arterial thromboembolism, and disseminated intravascular coagulation [10]. Both symptomatic and asymptomatic TEC are strongly associated with increased mortality rates. Therefore, it is essential to actively research methods to prevent and treat these potentially life-threatening complications [11].

The pathogenesis of thrombosis in PC is complex and involves tumor-derived procoagulant factors such as tissue factor (TF) and inflammatory cytokines. This hypercoagulable state is a result of both the tumor and the microenvironment (TME) surrounding it. TME comprises cancer-associated fibroblasts (CAFs), platelets, endothelial cells, tumor-associated macrophages (TAMs), and neutrophil extracellular traps (NETs), all of which encourage coagulation through separate mechanisms [12,13]. TEC in PC, particularly VTE, has significant implications for patient outcomes and survival. Various studies have highlighted the influence of VTE on progression-free survival (PFS) and overall survival (OS) in PDAC patients. Patients who developed VTE showed shorter median PFS and OS, which indicates a negative prognostic impact [4]. A decrease of around 35% in median OS and 26% in median PFS was observed for PC patients who developed VTE while receiving gemcitabine plus nab-paclitaxel chemotherapy [14]. Especially early and frequent onset of VTE after PDAC diagnosis was associated with a significant decrease in PFS and OS. In addition, symptomatic VTE events were linked to higher mortality compared to asymptomatic VTE cases [15]. That is why VTE is an independent negative prognostic factor, which should be assessed in high-risk patients. Moreover, individualized thromboprophylaxis is needed.

**Figure 1:**
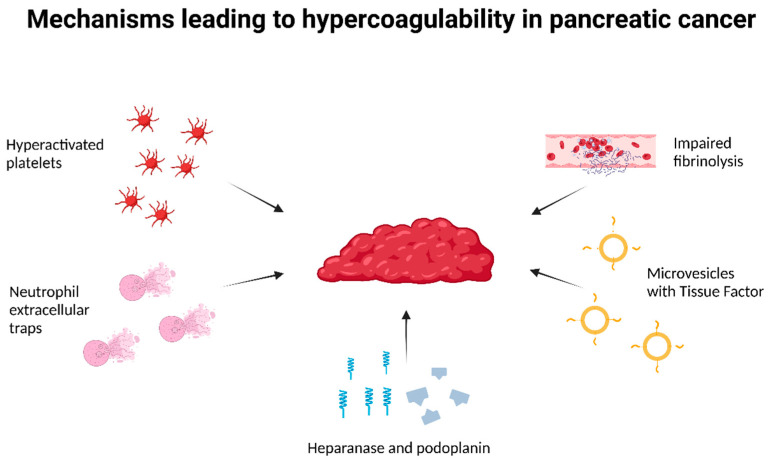
Schematic representation of the main mechanisms contributing to thromboembolism in PC. Tumor cells promote platelet activation and aggregation directly through thrombin and TF expression, and indirectly via microvesicles carrying procoagulant factors. Overexpression of TF, heparanase, and PDPN enhances the procoagulant state, while increased PAI-1 suppresses fibrinolysis. NETs and inflammatory cytokines further amplify coagulation and tumor progression. Based on [11,13,16,17,18,19,20,21]. Abbreviations: TF-tissue factor; PDPN-podoplanin; PAI-1- plasminogen activator inhibitor-1; NETs- Neutrophil extracellular traps. Created in BioRender. Sekuła, M. (2025); https://BioRender.com/i7q8gul.

The aim of the study is to present the underlying pathomechanism of TEC in patients diagnosed with PC. Increased knowledge in this field facilitates the more efficient identification of risk factors and the selection of patients at the highest risk of developing such complications. In addition, we review the spectrum of clinical manifestations of TEC and a specialized diagnostic approach. The unsatisfactory prognosis of PC patients with TEC highlights the challenge of selecting the most suitable preventive and therapeutic approach and emphasizes the importance of exploring the optimal management strategy (Figure 1).

## 2. Mechanisms Leading to Hypercoagulability in Pancreatic Cancer

The key molecular pathways and cellular interactions contributing to pancreatic cancer-associated thrombosis are summarized in Figure 1.

### 2.1. Role of Platelets

Interactions between tumor cells and platelets, causing platelet hyperactivation, play a crucial role in establishing a prothrombotic state during PC. Several studies have observed that platelet activation biomarkers, such as soluble P-selectin, thrombospondin-1, β-thrombospondin, and soluble CD40 ligand are elevated in patients with PDAC compared to their levels in healthy individuals. P-selectin and thrombospondin-1 are released by activated platelets upon direct interaction with tumor cells, promoting tumor-platelet aggregation and microthrombus formation [19,21]. Patients with PDAC also exhibit elevated platelet factor 4 (PF4) levels, which may serve as a prognostic biomarker, high PF4 serum levels are associated with a higher risk of venous thrombosis and poorer survival rates [21,22]. Platelet hyperreactivity was also observed in PDAC patients, manifesting as platelet degranulation in a shorter time frame than in healthy patients. Activated degranulating platelets release VEGF, which is responsible for inducing angiogenesis, a mechanism utilized by tumors to promote growth. Additionally, PDGF is released, whose signaling pathway plays a crucial role in the progression of PC [9,23,24,25].

The activation of the platelets in patients with PDAC can occur either directly through the expression of procoagulant factors on the surface of tumor cells or indirectly, through the production of microvesicles (MVs) by tumor cells [26].

Thrombin-mediated platelet activation represents a significant amplifying step in PC-associated thrombosis. In PDAC, cancer cells express thrombin on their cell membrane, which activates platelets through protease-activated receptors (PAR) and interactions with coagulation factors V, VIII, XI, and XIII. Through PAR-1/PAR-4 signaling, thrombin activates platelets, causing rapid degranulation and the release of procoagulant mediators, such as P-selectin, phosphatidylserine-exposing platelet microparticles, vascular endothelial growth factor (VEGF), and platelet-derived growth factor (PDGF). These thrombin-activated platelets promote fibrin production, significantly increase tumor-platelet aggregation, and intensify the hypercoagulable state characteristic of PC [20,21,23].

MVs secreted by PDAC cells contain factors that exhibit prothrombotic effects, such as TF, fibrin, and podoplanin (PDPN), and activate platelets indirectly [20]. The circulation of MVs in the blood is one of the hypotheses explaining why thrombosis occurs mainly at distant sites from the tumor [21].

### 2.2. Tissue Factor

TF, the transmembrane receptor activating the extrinsic coagulation pathway, plays a major role in hypercoagulability in PC. Under normal circumstances, it is absent in the endothelium of pancreatic vessels and only present in smooth muscle cells surrounding them [10]. On the contrary, in cancerous growths, TF is a common finding among the cells and seems to coexist with minimal expression of tissue factor pathway inhibitor (TFPI) [23]. It is expressed in various cancers, with high prevalence (over 75% of cases) in PC, cervical cancer, and head and neck squamous cell carcinoma [27]. On the contrary, its low expression was found in breast, ovarian, and bladder cancer, which is in discordance with previous reports [27,28]. The high TF expression is also more prevalent in metastatic tumors found in other organs, such as the lungs and colon, and is associated with worse clinical outcomes [17,29]. In PC, increased TF expression strongly correlates with high histological grade [18], and TF expression increases with disease progression and is associated with the presence of metastasis [30,31].

Animal studies have shown that tumors with overexpression of TF are associated with increased mitogenic activity of endothelial cells, enhanced transcription of VEGF, and decreased expression of thrombospondin-2, exhibiting proangiogenic properties [32]. Cancer-associated venous thromboembolism mediated by TF is thought to be caused by several mechanisms, including the secretion of TF-carrying MV by cancerous cells, the secretion of MV by immune cells, and the circulation of TF-positive cancer cells in vessels [26]. The level of TF in blood plasma (100 pg/mL or higher) in patients with PC seems to be predictive of cancer-associated thromboembolism, with positive and negative predictive values of 23.1% and 94.6%, respectively [33]. An important regulatory element of this TF-driven cascade is TFPI, the main endogenous inhibitor of the TF-FVIIa complex. By inhibiting both TF-FVIIa and FXa, TFPI limits excessive thrombin generation. Nevertheless, TFPI expression within the tumor microenvironment in PC is significantly reduced, creating an imbalance favoring a persistent procoagulant state. Furthermore, TFPI can interact with heparinase to displace it from endothelial surfaces, which further reduces its inhibitory ability and increases the production of TF-dependent thrombin. Therefore, TF-mediated hypercoagulability is enhanced by the loss of TFPI regulatory control, which contributes to the increased incidence of thrombosis seen in PC patients [19,22,23]. Moreover, in animal models, the secretion of TF-positive MVs promotes cancer-cell clustering, mediated by fibrin formation, and adhesion of cancerous cells to distant sites, which facilitates metastatic potential [24,25].

### 2.3. Heparanase

Heparanase is an enzyme of the β-glucuronidase family, which can catalyze the hydrolysis of the β-bond between glucuronic acid and the N-acetylglucosamine residue in heparan sulfate chains, which are part of heparan sulfate proteoglycans. In this manner, heparanase contributes to the remodeling of the extracellular matrix. Heparanase plays a crucial role in physiological processes, including wound healing, as well as in pathological conditions such as cancer and inflammatory diseases [34].

Elevated levels of heparanase have been observed in many types of cancer, including PC [20]. The overexpression of heparanase is associated with a higher risk of metastasis and indicates a poor prognosis [35]. It has been suggested that heparanase participates in cancer metastasis by damaging heparan sulfate-containing extracellular matrix structures in the basement membrane, facilitating cancer cell invasion [19].

Heparanase also exhibits actions independent of its enzymatic activity; for example, in tumors, it promotes leukocyte adhesion and migration by endothelial cells, induces VEGF expression, and participates in lymphangiogenesis [36]. In a non-enzymatic mechanism, heparanase also exhibits procoagulant activity through interaction with TFPI. Heparanase interacts with membrane-bound TFPI, causing it to dissociate and, consequently, increasing cell surface procoagulant activity. Heparanase also upregulates TF by enhancing its expression and directly increasing its activity, thereby increasing the factor Xa level and activating the coagulation cascade [22]. Furthermore, in PC, heparanase promotes tumor growth and chemoresistance by enhancing insulin receptor signaling and GLUT-4-mediated glucose transport to tumor cells [37].

### 2.4. Fibrinolysis

Although cancers are primarily associated with hypercoagulation effects, there is also an association between factors responsible for fibrinolysis and tumor growth and progression. Studies suggest that fibrin and fibrinogen degradation fragments contribute to proliferation, endothelial cell migration, cytokine expression, and differentiation, which are crucial cancer features [38].

However, PC is associated with greater procoagulant than fibrinolytic activity, with multiple mechanisms responsible for hypercoagulability [5]. PC cells have been shown to express plasminogen activator inhibitor type 1 (PAI-1), which inhibits the fibrinolytic process. Increased levels of PAI-1 in plasma are connected to a higher risk of thrombotic events. In patients with PC, mean plasma PAI-1 activity was found to be 15.3 ± 8.9 U/mL, significantly higher than the 6.6 ± 4.2 U/mL observed in healthy controls (*p* < 0.001), supporting its role in increased thrombotic risk [39]. Another crucial regulator of fibrinolysis, which is the principal physiological inhibitor of plasmin, is Alpha 2-antiplasmin. It prevents premature fibrin degradation by binding and inactivating free plasmin, therefore stabilizing thrombus structure. Increased incorporation of Alpha 2-antiplasmin into fibrin networks and heightened cross-linking mediated by factor XII enhance resistance of clots to lysis, further contributing to the hypofibrinolytic state in PC patients [21,40,41].

### 2.5. Podoplanin

PDPN is a transmembrane glycoprotein typically expressed in kidney podocytes, alveolar type I cells, osteocytes, basal keratinocytes, mesothelial cells, and lymphatic endothelial cells [5]. However, its overexpression has been found in transformed cells, CAFs, and inflammatory macrophages [42]. PDPN expression in CAFs has been shown to correlate with lymphatic and vascular invasion, tumor size, and shorter survival time [43]. Animal studies on glioblastoma cells have shown that PDPN interacts with the platelet-receptor C-type lectin-like receptor 2, thereby activating it and causing its aggregation [44]. Additionally, PDPN is increased in circulating MVs in patients with PC throughout the body, contributing to systemic thrombophilia [40].

### 2.6. Neutrophil Extracellular Traps

Another factor contributing to hypercoagulability is the increased formation of NETs. NETs are a part of the immunological system and are responsible for protecting organisms against infection [41]. NETs are web-like structures composed of decondensed chromatin and associated cytosolic and granule proteins, including histones, neutrophil elastase, myeloperoxidase, and other antimicrobial molecules. Their formation primarily occurs through a specialized form of neutrophil cell death known as NETosis, which involves the breakdown of the nuclear envelope, chromatin decondensation, and the release of DNA and proteins into the extracellular space [41]. However, their activity often promotes thrombosis, primarily by occluding blood vessels [41] and indirectly activating platelets, coagulation factors, and thrombin, as well as promoting thrombin activation and fibrin deposition [38].

Tumor cells often express factors inducing NET formation [38]. In PC, the mechanism responsible for this phenomenon is the overexpression of tissue inhibitor of metalloproteinases-1 [21]. Additionally, inflammation, which is present in the state of cancer, also contributes to NET formation and cancer progression [21]. Notably, neutrophil activation markers, such as calprotectin, have been identified as predictors of VTE in PC and distal extrahepatic cholangiocarcinoma, highlighting the role of neutrophil activity in thrombotic risk assessment [45].

Despite the thrombotic effect, NETs are also responsible for tumor progression by capturing circulating tumor cells within small blood vessels, facilitating their entrapment and colonization in distant tissues. Activated neutrophils primarily release them during NETosis. While initially described as a host defense mechanism against pathogens, NETs also create a microenvironment conducive to tumor cell adhesion and survival [41].

### 2.7. Cytokines

Inflammatory infiltration and its associated secretions, including cytokines and leukotrienes, play a pivotal role in the tumor microenvironment, tumor development, and metastasis progression [46]. PC has been associated with markedly elevated cytokines levels, which might be associated with poor clinical outcome [47]. Among the more commonly secreted, by both PC and stromal cells, are tumor necrosis factor α (TNF-α), interleukin 6, and interleukin 8 (IL-8)—these are induced by both hypoxemia and inflammation caused by cancerous growth. IL-8 is commonly associated with chronic inflammation and the attraction of neutrophils, but it also upregulates the expression of VEGF and neuropilin-2 in PC cells [48]. Furthermore, the matrix metalloproteinases themselves induce hypercoagulability by interacting with endothelial cells and support metastasis by degrading the extracellular matrix, which also increases NET formation [49]. TNF-α indirectly contributes to the anti-apoptotic activity of PC cells and causes poor clinical outcomes for patients, such as weight loss and cachexia [50,51]. These inflammatory modulators increase TF expression, downregulate TFPI, and promote PAI-1 synthesis, directly contributing to acquired thrombophilia [52]. Additionally, the inflammatory response within the endothelial wall reduces levels of thrombomodulin, an activator of the strong anticoagulant protein C pathway, and alters the expression profile of adhesion molecules, thus enabling the migration of leukocytes and platelets [53]. Chemotherapy and radiation may also cause endothelial injury, further increasing the risk of thrombosis. Activated monocytes in cancer patients show increased levels of TFs, while polymorphonuclear leukocytes drawn to the cancer site release reactive oxygen species and proteases that further damage the endothelium [22,54]. Overall, the immune response and tumor-mediated changes injure the endothelium, enhance tissue factor activity, impair natural anticoagulant pathways, and create a sustained prothrombotic environment.

## 3. Risk Factors

To organize the risk factors of TEC in PC, they can be grouped into individual patient-, tumor-, and therapy-dependent factors (Table 1).

### 3.1. Patient-Dependent Risk Factors

The first group of risk factors includes poor Eastern Cooperative Oncology Group performance status, female sex, older age, body mass index (BMI) ≥ 25 kg/m^2^, a D-dimer level > 1.2 µg/mL, and a hemoglobin (Hb) level < 10 g/dL [9,55]. Previous thromboembolic events are also associated with an increased risk of subsequent VTE episodes [56]. The influence of genetic factors on the risk of TEC may be attributed to differences in the incidence of thromboembolic events across various populations. A higher incidence of TEC was observed in the Western population relative to the Asian one. The G20210A variant of the thrombin gene, more commonly present in the Western population, causes hypercoagulability and correlates with an increased prevalence of thromboembolism [57]. Mutations in the KRAS and p53 genes are other genetic factors associated with TEC in PC patients. Activation of the KRAS oncogene promotes a prothrombotic phenotype through upregulation of TF and the release of TF-positive microparticles from tumor cells, which activate the extrinsic coagulation cascade. Loss of *TP53* function further enhances this effect by diminishing transcriptional control over procoagulant and inflammatory mediators, including TF and P-selectin, leading to increased thrombin generation and fibrin formation. These molecular changes amplify the systemic hypercoagulable state typical of PC-associated thrombosis [58,59,60,61].

### 3.2. Cancer-Dependent Risk Factors

The presence of distant metastasis is a crucial risk factor for TEC occurrence in PC. In the California Cancer Registry study, which included over 6500 patients with PC, patients with metastatic disease had a 3.3-fold increase in the risk of developing VTE, compared to patients with regional disease [62]. In the BACAP-VTE study, conducted on over 700 patients with PC, the risk of developing VTE was 2.5-fold higher in patients with metastatic disease, compared to patients with resectable tumors [4]. Tumor location is also clinically relevant. PC located in the neck, body, or tail of the pancreas is associated with an approximately twofold higher risk of thromboembolism compared with head-localized tumors [4,57]. Non-head PC usually presents symptoms later and is therefore diagnosed at a more advanced stage, which is associated with an increased incidence of TEC [57]. Advanced disease is frequently accompanied by cancer cachexia, a multifactorial syndrome characterized by ongoing loss of skeletal muscle mass that cannot be reversed by conventional nutritional support and that leads to functional impairment [63]. PC has one of the highest prevalences of cachexia, affecting approximately 63–64% of patients [64]. This is primarily caused by systemic inflammation driven by increased cytokine levels, impaired exocrine and endocrine pancreatic function, and alterations in the intestinal tract, with a particular impact on the microbiome [65]. Progressive cachexia limits mobility, which further increases the risk of TEC [57].

### 3.3. Therapy-Dependent Risk Factors

The therapeutic management of PC associated with an increased risk of TEC is chemotherapy. Therapy regimens particularly associated with TEC include gemcitabine, paclitaxel, oxaliplatin, and 5-FU [66,67]. In addition, the administration of chemotherapy through a central catheter increases the risk of thrombotic complications [55]. Cancer patients undergoing resection surgery have a significantly higher risk of VTE complications compared to non-cancer surgical patients, with large prospective data showing approximately twice the incidence in oncologic surgery [68]. In pancreatic cancer, this risk remains prolonged after surgery, particularly following neoadjuvant therapy [69]. An increased incidence of VTE in patients undergoing surgical treatment for PC is associated with BMI ≥ 30 kg/m^2^, previous anticoagulant treatment, recurrence of disease, and patient immobilization longer than 3 days [68,69].

### 3.4. Risk Stratification of Thromboembolism in Cancer

Various generally accepted scales are used to assess the risk of TEC in cancer patients. In their study, R. Wollems et al. collected scales that can help assess the risk of venous thrombosis in oncological patients. Each scale (Khorana Score, Vienna Model, ONCOTHROMB, Li Model, ONKOTEV) includes cancer type as a risk factor. Other factors most frequently mentioned in the above scales include BMI, cancer stage, Hb levels, use of red blood cell (RBC) growth factors, white blood cell (WBC) count, and platelet (PLT) count. In the cohort, anemia, thrombocytosis, and leukocytosis were prevalent among patients who developed postoperative VTE, highlighting the pro-inflammatory and hypercoagulable state characteristic of PC. The inclusion of D-dimer distinguishes the Vienna Model. ONCOTHROMB additionally includes genetic germline mutations such as rs4524, rs6025, rs2232698, rs2227631, rs268, rs169713, rs11696364, rs5110, and rs6003. The Li Model assesses immobilization, while ONKOTEV scores vascular and lymphatic compression; both scales incorporate previous VTE [70]. Another assessment model, the PROTECHT score, includes Hb, use of RBC growth factors, WBC and PLT count, BMI, and use of gemcitabine or platinum-based therapy [71].

In the recent prospective BACAP cohort, which included 760 patients with pancreatic cancer receiving outpatient chemotherapy, the Khorana, PROTECHT, and ONKOTEV scores demonstrated poor predictive performance. The time-dependent concordance indices ranged from 0.50 to 0.53, indicating no better discrimination than chance in predicting 6-month VTE risk [72]. These results suggest that current risk scores have limited value for risk stratification in pancreatic cancer and highlight the need for more accurate, disease-specific prediction models. In accordance with the ITAC and ESMO clinical practice guidelines, cancer stage should be given greater consideration when assessing VTE risk in this patient population [73,74].

## 4. Symptoms

Clinically, common manifestations of VTE primarily include PE and DVT, both of which are life-threatening conditions. Symptoms of TEC in PC can vary depending on the affected location [75]. A significant number have experienced DVT and/or PE without showing any symptoms. About 3% of patients undergoing computed tomography angiography for reasons other than suspected PE are incidentally found to have a pulmonary artery thrombus. Cancer patients are at higher risk of asymptomatic VTE in the abdominal area, which is detected in 2–5% of patients undergoing abdominal computed tomography (CT) scans [76]. Recognizing the symptoms presented in Table 2 is crucial for prompt diagnosis and appropriate management of TEC in PC patients. Early intervention can help prevent serious consequences associated with these conditions.

## 5. Diagnosis

Diagnosis of thromboembolic events in patients with PC requires a specialized approach. A normal d-dimer result helps to exclude PE, but the value of the test in PC patients may not be very reliable and requires verification by additional diagnostic tools. Because baseline levels are often higher in cancer patients than in non-cancer individuals, d-dimer has a lower specificity for VTE [80,81]. D-dimer elevation indicates not only the presence of acute thrombosis but also the concomitant activation of coagulation caused by tumor-related procoagulant activity and systemic inflammation [82,83]. Even in the absence of detectable thrombus, significantly elevated d-dimer values may occur because tumor biology itself can increase fibrin turnover independently of overt VTE [84]. While positive results are frequent and require imaging confirmation, d-dimer testing for cancer has limited specificity, meaning it only retains its rule-out value in individuals with low clinical probability. Meta-analyses in oncology populations show that using the standard manufacturer cutoff (0.5 mg/L) yields very high sensitivity (96.4%) but extremely low specificity (26.4%), allowing only 9% of cancer patients with low clinical probability to be ruled out without imaging. This finding confirms that the standard d-dimer cutoff is unreliable for excluding VTE in cancer and highlights the need for a meta-analysis focused exclusively on pancreatic cancer patients. As a result, it should never be interpreted alone but rather together with clinical probability scores [85,86,87]. Moreover, chronically elevated d-dimer levels in cancer patients correlate with a higher chance of recurrent VTE and worse overall survival, which emphasizes their prognostic rather than diagnostic role [88,89]. In cancer patients, DVT diagnosis relies on compression ultrasonography, while PE is diagnosed using CT pulmonary angiography [76].

Early and accurate diagnosis is crucial for optimizing patient outcomes and implementing appropriate management strategies. Some studies report that approximately 52% of VTE cases among PC patients are incidentally diagnosed, which highlights the importance of thorough diagnostic approaches [15].

## 6. Treatment

**Figure 2:**
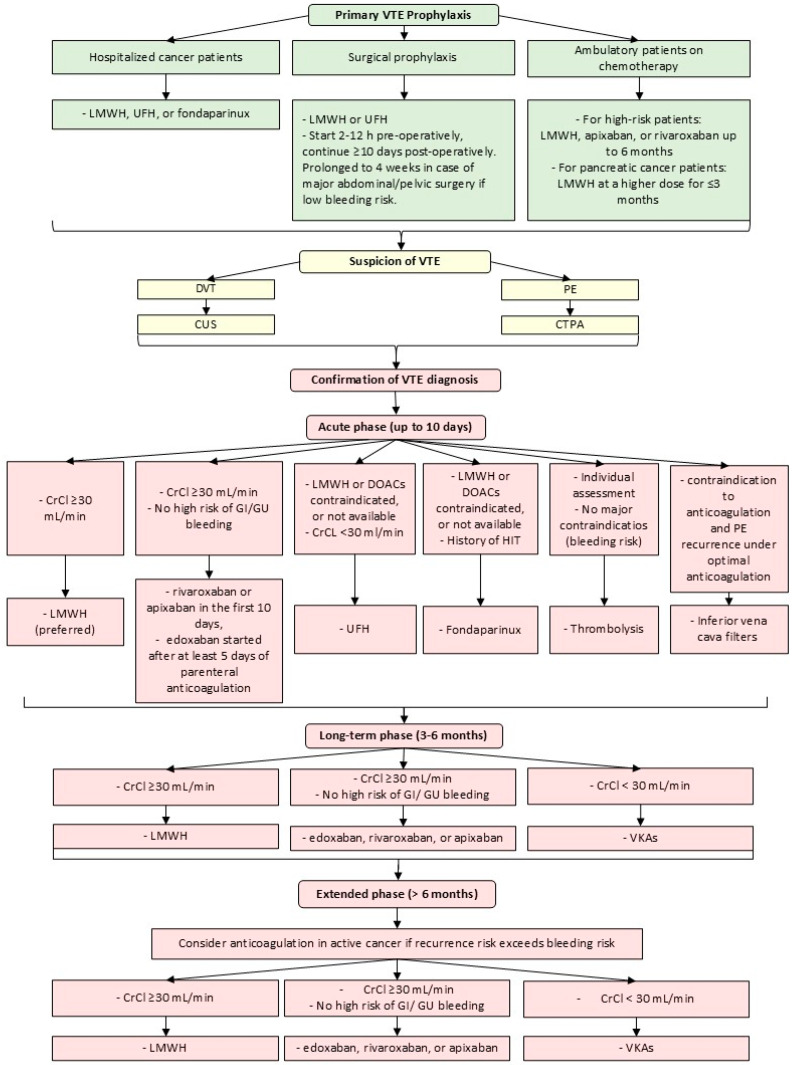
A Clinical Management Algorithm in Cancer Patients. Abbreviations: VTE—venous thromboembolism; LMWH—low-molecular-weight heparin; UFH—unfractionated heparin; DVT—deep venous thrombosis; CUS—compression ultrasonography; PE—pulmonary embolism, CTPA—CT pulmonary angiography; CrCl—creatinine clearance; GI—gastrointestinal; GU—genitourinary; VKAs—vitamin K antagonists.

Advances in cancer therapy and improved overall survival have led to a growing population of patients living longer with active malignancy. As a result, the cumulative risk of thromboembolic disease in cancer patients is increasing, particularly in those with comorbidities such as diabetes, obesity, dyslipidemia, or hypertension [56,73]. A nested case–control study found that certain medications are strongly linked to an increased risk of VTE. These include diabetes drugs such as insulin and sulphonylureas, the loop diuretic furosemide, glucocorticoids, gabapentinoids, and psychotropic medications [90]. This elevates the number of patients in the cancer population who may benefit from anticoagulant therapy. Various guidelines distinguish between the treatment of acute, long-term, and extended phases of cancer-associated thrombosis in oncology patients (Figure 2) [74].

### 6.1. Acute Phase Treatment

In the acute phase, low-molecular-weight heparin (LMWH) is a preferable initial treatment in the first 5–10 days. The advantage of LMWH is particularly emphasized in patients with a creatinine clearance (CrCl) of ≥30 mL/min, especially in patients with gastrointestinal cancers, due to the reduced risk of bleeding complications [74]. The European Society for Medical Oncology (ESMO) recommends taking LMWH once or twice daily, while the International Initiative on Thrombosis and Cancer (ITAC) guidelines prefer one dose per day. According to ITAC guidelines, when a twice-daily regimen is necessary, only enoxaparin is recommended [73,74]. Unfractionated heparin (UFH) or fondaparinux remains an alternative treatment option [74]. UFH is preferred in patients with CrCl < 30 mL/min, whereas fondaparinux may be considered in those with a prior history of heparin-induced thrombocytopenia [73,74]. In selected, life-threatening cases, inferior vena cava filters may be considered during the initial management of VTE, when anticoagulation is contraindicated. Contraindications to anticoagulation should be reassessed regularly, and anticoagulant therapy should be administered when it is considered safe [73].

### 6.2. Long-Term and Extended Treatment

LMWH has been shown to be more effective than vitamin K antagonists (VKAs) in the long-term phase treatment, specifically the first 3–6 months after diagnosis [74].

An alternative to LMWH for long-term therapy is the administration of direct oral anticoagulants (DOACs)—apixaban, edoxaban, rivaroxaban [74]. Relative to LMWH, they are even more effective in preventing recurrent VTE, have a more convenient form of administration, but also a higher rate of hemorrhagic complications, especially in gastrointestinal cancers [91,92]. Moreover, they can be administered when the CrCl is ≥30 mL/min [74]. Therefore, the benefits, disadvantages, and patient preferences should be assessed. Anticoagulant treatment should last at least 6 months [74].

In patients with active cancer, if the risk of recurrence outweighs the risk of bleeding, anticoagulation treatment may be prolonged > 6 months with LMWH, apixaban, edoxaban, rivaroxaban, or VKAs [73].

### 6.3. Management of Recurrent VTE

If VTE recurs, the ITAC guidelines recommend considering either a 20–25% increase in the LMWH or a switch to DOACs. If recurrence occurs during DOAC therapy, treatment with LMWH is preferred. In patients receiving VKAs, switching to LMWH or DOACs should be considered [74]. Inferior vena cava filter implantation is a non-pharmacological treatment option for TEC. It is indicated as an adjunct to anticoagulant therapy in patients with recurrent VTE or progressive thrombosis despite optimal anticoagulation, and in those with an absolute contraindication to anticoagulant therapy [73,93].

### 6.4. Emerging Anticoagulant Strategies: Factor XI Inhibitors

Recent studies are exploring potentially more effective anticoagulation therapies in cancer patients that will have the best possible safety profile. Factor XI (FXI) of the intrinsic coagulation pathway is considered essential for the initiation of thrombosis but may be dispensable for hemostasis [94]. The four FXI inhibitors (an antisense oligonucleotide—fesomersen, small molecule FXIa inhibitors—milvexian, and monoclonal antibodies—osocimab and abelacimab) demonstrated at least equal efficacy to enoxaparin in randomized controlled trials conducted on surgical patients, respectively FXI-ASO TKA study (NCT01713361), FOXTROT study (NCT03276143), EudraCT No. 2019-003756-37, and NCT03891524 [95,96,97,98]. Abelacimab appears to have a favorable pharmacological profile and does not interfere with clinically relevant hemostasis, making it a promising anticoagulant whose efficacy and safety require further study. Currently, two ongoing phase III trials are assessing this drug in comparison to standard of care therapy in patients with CAT (Table 3).

## 7. Prophylaxis

Thromboembolism influences increasing mortality in patients with PC. Therefore, prevention of thromboembolic incidents seems to be relevant for this patient population. The disease’s course alters the risk of TEC, so the balance of the benefits of anticoagulant prophylaxis and the risk of bleeding should be regularly reassessed based on the patient’s condition and current therapy. Before surgery, oncology patients should be assessed for their individual risk factors for developing TEC. In addition, when considering the inclusion of perioperative prophylaxis, attention should be paid to the type, duration of the procedure, and any contraindications to pharmacological thromboprophylaxis.

According to ESMO guidelines, LMWH or UFH is the standard of prophylaxis in surgical patients with a high risk of TEC and a low risk of bleeding [73]. LMWH prophylaxis was not found to be more effective than UFH, but it is often preferred for oncological patients because it carries a lower risk of heparin-induced thrombocytopenia and less frequent wound hematoma formation [73,74]. As an alternative option, the administration of fondaparinux can be considered [73]. American Society of Clinical Oncology (ASCO) recommendations include rivaroxaban or apixaban in prophylactic doses as an alternative to LMWH, after the initial use of LMWH or UFH [93]. Bedridden or hospitalized oncology patients should take prophylactically: LMWH, UFH, or fondaparinux [73,74]. The addition of mechanical thromboprophylaxis, such as intermittent pneumatic compression or graduated compression stockings, more effectively protects extremely high-risk patients and may be a viable solution when pharmacological prophylaxis is contraindicated; however, it should not be used as standard monotherapy [93].

The timing of prophylaxis administration influences its anticoagulant efficacy. Pre-operative prophylaxis (administered 2–12 h before surgery) has better anticoagulant effects compared to post-operative prophylaxis and carries a similar risk of bleeding [73,74]. It is recommended to use the highest prophylactic dose of LMWH once daily or UFH three times daily and continue for at least 7–10 days after surgery [73,93]. In cases of major abdominal surgery, when the patient is not at high risk of bleeding, prolonged prophylaxis for 4 weeks can be used [74,93].

The appropriateness of including and selecting the type of anticoagulant prophylaxis in non-surgical patients is the subject of active debate and numerous clinical trials. The CONKO-004 trial conducted on outpatients with histologically confirmed advanced PC shows the benefit of enoxaparin over placebo in primary thromboprophylaxis in PDAC. Within 3 months, symptomatic VTE occurred in 2 of 160 (1%) patients taking prophylactic doses of enoxaparin and in 15 of 152 (10%) patients in the placebo group (*p* = 0.001) [99]. The FRAGEM trial compared the rates of VTE in patients with advanced PC treated with 1000 mg/m^2^ gemcitabine (GEM) versus patients treated with gemcitabine with weight-adjusted dalteparin (GEMWAD) for 12 weeks. In the group treated with the GEMWAD regimen, 2 out of 59 (3%) patients developed VTE, compared to 14 out of 60 (23%) with GEM alone (*p* = 0.002). Both studies showed no significant increase in bleeding [100]. The CASSINI trial included PC patients at high risk of VTE (Khorana score ≥ 2) initiating a new treatment regimen. The study compared the efficacy of a prophylactic dose of rivaroxaban (10 mg) to a placebo administered orally once a day. During the intervention period, 5 of 135 (3.7%) study members taking rivaroxaban experienced TEC, compared to 14 of 138 (10.1%) taking placebo (*p* = 0.034) [101]. These three trials are compared in Table 4.

The COMPASS-CAT and Vienna-CATS scales may provide a certain diagnostic value for VTE in cancer patients, identifying patients at risk of VTE [102,103]. According to the ESMO guidelines, cancer patients at high risk of thrombosis, which means above 8–10% at 6 months, can take prophylactic doses of LMWH, apixaban or rivaroxaban for up to 6 months. Ambulatory PC patients receiving first-line therapy may undergo prophylaxis with LMWH at a higher dose (150 IU/kg dalteparin or 1 mg/kg enoxaparin) for a maximum of 3 months [73]. In accordance with ASCO guidelines, the administration of LMWH, direct factor Xa inhibitor, or VKA may be extended beyond 6 months in patients at high risk of thrombosis who have metastatic disease or are receiving chemotherapy [93]. The ITAC guidelines in outpatients with locally advanced or metastatic PC treated with systemic chemotherapy and who are at low risk of bleeding recommend prophylaxis with LMWH or DOACs (rivaroxaban or apixaban). It should be noted that if the platelet count is <80 × 10^9^ per L or the patient has obesity, proper assessment of using pharmacological prophylaxis is needed (Table 5) [74].

## 8. Conclusions

Pancreatic cancer is one of the most lethal malignancies, and its rising incidence, along with frequent thromboembolic complications, further worsens patient outcomes. PDAC-related thrombosis is triggered by the interaction between cancer cells and the prothrombotic tumor microenvironment. A network of cancer cells, fibroblasts, platelets, macrophages, neutrophils, and endothelial cells promotes initiation of the extrinsic coagulation pathway, platelet activation, microthrombus formation and fibrin deposition. The correlation of clinical symptoms with laboratory and imaging findings, as well as risk factors, enables a quick diagnosis, which is essential for administering appropriate treatment and improving prognosis. The present management does not provide sufficient care for patients with PC, thus emphasizing the necessity to gain knowledge about TEC and to explore and implement novel preventive and therapeutic strategies.

Limitations: A limitation of this review is that the literature was chosen by the authors, which may have introduced selection bias, even though attempts were made to include the most significant and recent studies.

## Figures and Tables

**Table 1:** Risk factors of TEC in PC patients.

Risk Factor Category	Examples of Risk Factors
Patient-related	poor Eastern Cooperative Oncology Group performance status
	female sex
	older age
	BMI ≥ 25 kg/m^2^
	D-dimer level > 1.2 µg/ml
	Hb level < 10 g/dL
	previous thromboembolic events
	Western population (G20210A variant of the thrombin gene)
	mutations in the KRAS and p53 genes
Tumor-related	stage IV of the tumor
	non-head location of PC
	cancer cachexia
Therapy-related	chemotherapy
	central catheter
	surgical treatment

Abbreviations: BMI—body mass index; Hg—hemoglobin; KRAS—Kirsten rat sarcoma viral oncogene homolog; PC—pancreatic cancer.

**Table 2:** Characteristics and incidence of TEC in PC patients.

Complications	Incidence in PC Patients	Characteristics
Lower and Upper Extremes VTE [7]	26%	Oedema
		Erythema
		Paresthesia
		visible enlargement of veins in the area
		excessive warmth
		increased extremity girth
Lower Extremes VTE [7]	26%	calf tenderness
		lower limb pain
Upper Extremes VTE [7]		pain in the shoulder and axilla
		pain radiating to lower jaw, head, neck
Pulmonary Embolism [7]	17%	common symptoms:
		tachycardia,
		tachypnea,
		dyspnea,
		pleural pain,
		cough
		less common symptoms:
		increased body temperature,
		hemoptysis,
		collapse,
		fainting
Migratory Thrombophlebitis (Trousseau Syndrome) [77]	6.9%	atypical superficial vein locations, such as the upper extremities and the chest
		episodes of spontaneous regression and recurrences
		resistance to antithrombotic treatment
Visceral Vein Thrombosis [36]	16.7%	location in portal vein, splenic vein, or mesenteric vein
		abdominal pain,
		splenomegaly,
		esophageal varices,
		ascites
Hepatic Vein Thrombosis (Budd-Chiari Syndrome) [78]	3.5%	discomfort in the abdomen,
		ascites,
		hepatomegaly
		abdominal pain
Arterial Thromboembolism [36]	5.9%	myocardial infarction
		ischemic stroke
Marantic Endocarditis [79]	rare	new-onset heart murmurs

Abbreviations: VTE—venous thromboembolism.

**Table 3:** Studies comparing Abelacimab to standard anticoagulant therapy in the treatment of cancer-associated VTE.

Trial Name/ID	Phase	Population/Indication	Intervention vs. Comparator	Primary Efficacy Endpoint	Secondary Efficacy Endpoint
ANT-007 (ASTER) NCT05171049	III	CAT in patients for whom DOAC treatment is recommended	Abelacimab vs. Apixaban over a 6-month treatment	Recurrence of VTE	Composite of MB + CRNMB
ANT-008 (MAGNOLIA) NCT05171075	III	CAT in patients with GI or GU cancer	Abelacimab vs. Dalteparin (LMWH)	Recurrence of VTE	Composite of MB + CRNMB

Abbreviations: CAT—cancer-associated thrombosis; VTE—venous thromboembolism; MB—major bleeding; CRNMB—clinically relevant non-major bleeding; GI—gastrointestinal; GU—genitourinary; LMWH—low-molecular-weight heparin.

**Table 4:** Comparison of trials CONKO-004, FRAGEM, and CASSINI.

Trial	Study Population	Intervention	Control	Duration	VTE Incidence	Bleeding Events	Key Findings	Reference
CONKO-004	312 patients with advanced PDAC	Enoxaparin (prophylactic dose)	Placebo	3 months	2/160 (1%) vs. 15/152 (10%); *p* = 0.001	No significant increase in bleeding	Enoxaparin significantly reduced symptomatic VTE compared to placebo	[99]
FRAGEM	119 patients with advanced PC	Gemcitabine + weight-adjusted dalteparin (GEMWAD)	Gemcitabine alone (GEM)	12 weeks	2/59 (3%) vs. 14/60 (23%); *p* = 0.002	No significant increase in bleeding	Dalteparin prophylaxis reduced VTE rate without increasing bleeding risk	[100]
CASSINI	273 patients at VTE risk, starting new therapy	Rivaroxaban 10 mg daily (prophylactic dose)	Placebo	Intervention period	5/135 (3.7%) vs. 14/138 (10.1%); *p* = 0.034	-	Rivaroxaban lowered thromboembolic complications in high-risk patients	[101]

Abbreviations: PDAC—Pancreatic ductal adenocarcinoma; VTE—venous thromboembolism.

**Table 5:** Comparison of ESMO, ASCO, and ITAC guidelines.

Clinical Aspect	ESMO 2023	ASCO 2023	ITAC 2022	Summary
Initial (acute) treatment	LMWH preferred (first 5–10 days); UFH or fondaparinux as alternatives. DOACs (rivaroxaban or apixaban) are acceptable; edoxaban only after ≥ 5 days of parenteral therapy.	LMWH preferred for the first 5–10 days; UFH, rivaroxaban, or fondaparinux as alternatives.	Same approach; LMWH recommended as first-line. DOACs (rivaroxaban or apixaban); edoxaban after ≥ 5 days of parenteral anticoagulation.	LMWH preferred in acute phase.
Renal function	LMWH if CrCl ≥ 30 mL/min; UFH preferred if < 30 mL/min.	Comparable recommendations.	Comparable recommendations.	Dose adjustment based on renal function.
Long-term therapy (3–6 months)	LMWH or DOACs; LMWH preferred in luminal GI cancers.	LMWH or DOACs; VKAs acceptable if LMWH/DOACs unavailable.	-LMWH or DOAC for ≥6 months	DOACs accepted alternatives; caution in GI/GU cancers.
Extended therapy (>6 months)	Continue LMWH or DOAC if cancer remains active or recurrence risk > bleeding risk.	LMWH or DOACs for ≥ 6 months (up to 12 months in metastatic disease or during chemotherapy). VKAs are acceptable if others are not available.	LMWH or DOACs for ≥6 months, extendable to 12 months.	Continuation recommended in active cancer or high recurrence risk.
DOAC use (apixaban, edoxaban, rivaroxaban)	Effective, acceptable alternative to LMWH. ↑ bleeding risk in GI/GU cancers for edoxaban and rivaroxaban.	Edoxaban and rivaroxaban recommended for VTE treatment; apixaban and rivaroxaban for prophylaxis. Check for drug–drug interactions.	Effective ↑ bleeding risk in upper GI cancers.	DOACs preferred in low-bleeding-risk, non-GI tumors. LMWH preferred if CYP3A4 or P-gp interactions present.
When VTE recurs	Consider IVC filters.	Alternative anticoagulant regimen or increasing the dose of LMWH	↑ LMWH dose by 20–25% or switch between LMWH ↔ DOACs/VKAs.	ITAC provides the most detailed escalation recommendations.
IVC filters	Acute, life-threatening VTE with absolute contraindication to anticoagulation. Adjunct in recurrent or progressive VTE despite adequate therapy, or when anticoagulation is absolutely contraindicated.	Not recommended in chronic VTE (>4 weeks). Consider in acute VTE (<4 weeks) with absolute contraindication to anticoagulation. May be added in case of progression despite optimal therapy (weak recommendation).	Initial treatment: consider if anticoagulation contraindicated. Consider in PE with recurrence despite optimal anticoagulation. Not recommended for routine prophylaxis.	Restricted use; only in acute VTE with absolute contraindications or progression despite therapy.
Thrombocytopenia	Platelet count: >50 ×10^9^/L—full therapeutic dose >40–50 ×10^9^/L—consider full dose + platelet transfusion (high-risk VTE only) <25 ×10^9^/L—temporary discontinuation	Platelet count: <20 × 10^9^/L—absolute contraindication 20–50 ×10^9^/L—relative contraindication (individual assessment)	Treatment, platelet count: >50 × 10^9^/L—full dose <50 × 10^9^/L—individual decision Prophylaxis, platelet count: >80 × 10^9^/L—pharmacological prophylaxis <80 × 10^9^/L—case-by-case	Different platelet thresholds across guidelines; ESMO provides the most detailed risk-adapted strategy.
Prophylaxis (hospitalized cancer patients)	LMWH, UFH, or fondaparinux. DOACs not recommended.	LMWH, UFH, or fondaparinux	LMWH or fondaparinux. DOACs not recommended.	Consensus: LMWH or fondaparinux; DOACs not recommended.
Surgical prophylaxis	Start 2–12 h pre-operatively, continue ≥ 10 days post-operatively. Prolonged to 4 weeks in case of major abdominal/pelvic surgery if low bleeding risk.	Start pre-operatively (2–12 h depending on drug), continue for at least 7–10 days post-operatively. Prolonged to 4 weeks with LMWH in case of major abdominal/pelvic surgery for patients with high-risk features (e.g., obesity, restricted mobility, prior VTE), provided there is low bleeding risk.	Start pre-operatively (2–12 h depending on drug), continue for at least 7–10 days post-operatively. Prolonged to 4 weeks in case of major abdominal/pelvic surgery if low bleeding risk.	Full agreement across guidelines.
Ambulatory patients on chemotherapy	Prophylaxis for high-risk patients—an estimated risk of VTE > 8–10%. LMWH, apixaban, or rivaroxaban up to 6 months. For pancreatic cancer patients: LMWH at a higher dose for ≤3 months.	Prophylaxis for high-risk patients. LMWH, apixaban, or rivaroxaban up to 6 months.	Prophylaxis for patients with intermediate-to-high-risk of VTE.	Prophylaxis for high-risk patients based on individualized risk assessment.

Abbreviations: ASCO—American Society of Clinical Oncology; CrCl—creatinine clearance; DOAC—direct oral anticoagulant; DVT—deep vein thrombosis; ESMO—European Society for Medical Oncology; GI—gastrointestinal; GU—genitourinary; ITAC—International Initiative on Thrombosis and Cancer; IVC—inferior vena cava; LMWH—low-molecular-weight heparin; PE—pulmonary embolism; UFH—unfractionated heparin; VKA—vitamin K antagonist; VTE—venous thromboembolism.

## Data Availability

No new data were created or analyzed in this study. Data sharing is not applicable to this article.

## Abbreviations

The following abbreviations are used in this manuscript:

ASCO	American Society of Clinical Oncology
BMI	Body Mass Index
CAFs	Cancer-associated fibroblasts
CAT	Cancer-associated thrombosis
CrCl	Creatinine clearance
CRNMB	Clinically relevant non-major bleeding
CT	Computed tomography
DOACs	Direct oral anticoagulants
DVT	Deep venous thrombosis
EMT	Epithelial-to-mesenchymal transition
ESMO	European Society For Medical Oncology
FXI	Factor XI
GEM	Gemcitabine
FEMWAD	Gemcitabine with weight-adjusted dalteparin
GI	Gastrointestinal
GU	Genitourinary
Hb	Hemoglobin
IL-8	Interleukin 8
ITAC	International Initiative on Thrombosis and Cancer
IVE	Inferior vena cava
LMWH	Low-molecular-weight heparin
MB	Major bleeding
MVs	Microvesicles
NETs	Neutrophil extracellular traps
OS	Overall survival
PAI-1	Plasminogen activator inhibitor type 1
PAR	Protease-activated receptors
PC	Pancreatic cancer
PDAC	Pancreatic ductal adenocarcinoma
PDGF	Platelet-derived growth factor
PDPN	Podoplanin
PE	Pulmonary embolism
PFS	Progression-free survival
PF4	Platelet factor 4
PLT	Platelet
RBC	Red blood cell
TEC	Thromboembolic complications
TF	Tissue factor
TFPI	Tissue factor pathway inhibitor
TNF-a	Tumor necrosis factor a
UFH	Unfractionated heparin
VKA	Vitamin K antagonist
VTE	Venous thromboembolism
WBC	White blood cell
VEGF	Vascular endothelial growth factor
